# Rethinking personhood and agency: how AI challenges human-centered concepts

**DOI:** 10.3389/fpsyg.2025.1717828

**Published:** 2026-01-09

**Authors:** Lu Gao, Gustave Florentin Nkoulou Mvondo

**Affiliations:** 1 Department of Philosophy and Science, Southeast University, Nanjing, China; 2 College of Management, Shenzhen University, Shenzhen, China

**Keywords:** anthropomorphism, artificial intelligence, human-AI interaction, moral responsibility, perceived agency (intentional stance), personhood, sense of agency, sense of self

## Abstract

Artificial intelligence (AI) systems increasingly display behaviors once thought to be exclusively human, prompting people to attribute intention, emotion, and even moral responsibility to these agents. This philosophical perspective examines how psychology understand and respond to these developments in human-AI interaction: How and why do humans perceive and attribute personhood and agency to AI systems, what are the personal and social consequences of doing so, and what are the implications for psychology as a discipline? We thus advocate that psychology adopt an experience-focused approach to human-AI interactions, drawing primarily on psychological research related to the sense of agency and related aspects of self-experience. From this discussion, we develop a three-lens framework (explanatory, normative, and cultural) as a guide for advancing psychological research and practices related to human-AI interactions. We propose that psychology’s distinctive contribution in the age of AI is to analyze how concepts of agency and personhood function in lived human experience, particularly in shaping perceptions of control and responsibility regarding AI systems.

## 1 Introduction

Over the past 15 years, psychology has undergone profound societal and technological upheavals. Advances in neuroscience and genetics have reinforced its biological foundations (Baratta et al., 2022; Deary et al., 2022) while big data and computational modeling have transformed the study of behavior and cognition at scale. The replication crisis spurred a shift toward open science and more rigorous methodologies (Shrout and Rodgers, 2018), and global cultural shifts have challenged Western-centric assumptions, broadening psychology’s reach across diverse populations and contexts. The increasing need to address pressing issues regarding perceived agency and personhood in contemporary human-AI interactions presents a significant new challenge that psychology now faces.

In the past, misguided anthropomorphic attributions of intentionality, agency, sense of self, and personhood were relatively easy to recognize as errors. A kitchen chair that collapses when someone sits on it is not genuinely intending harm. The Sphex wasp may appear to engage in complex, intelligent behavior, such as checking for predators before entering its nest, but empirical work has shown that it operates according to a rigid, purely instinctual loop (Dennett, 2015, p. 11). Likewise, pets may display basic emotions and forms of intelligence, yet they are not ordinarily regarded as full persons or robust intentional agents. Until recently, other human beings were the only plausible candidates for personhood or genuine agency that most people encountered in everyday life, and machines or tools were not typically ascribed such statuses or treated as if they possessed them.

However, the world has changed. Our everyday routines and relationships are now increasingly shaped by interactions with AI systems, such as conversing with chatbots, utilizing autonomous navigation systems, and engaging with social robots (Wu and Mvondo, 2025; Zhao and McEwen, 2025). As AI systems display increasingly complex and apparently intelligent, “human-like” behaviors, drawing a clear line between tools and agential partners, and between persons and non-persons, has become increasingly blurred and uncertain. The perception of such systems is increasingly shifting away from seeing them as mere “tools,” and toward attributions of agency and even personhood.

Research shows that people frequently ask voice assistants for advice, thank navigation systems for safe arrival, and express sympathy toward robotic vacuums, often adopting an intentional stance that attributes beliefs, desires, and intentions to these systems (Sung et al., 2007; Verne et al., 2022; Marchesi et al., 2019; Dennett, 1989). People even report sadness or discomfort when humanoid robots fail at tasks or appear to be “hurt” (Morgante et al., 2024; von der Rosenthal- Pütten et al., 2013). Together, these findings suggest that humans increasingly relate to AI systems not merely as “tools,” but in ways normally reserved for other agents: beings with intentions, beliefs, desires, emotions, and at least implicitly in many cases, personhood.

One way to address these technological trends and human responses would be through engagement with the relevant philosophical questions. What is a person? What is an agent? Do any current AI systems count as persons or agents? Is it possible that some future AI systems might qualify? What moral consideration, if any, does AI deserve? Current human-AI interactions challenge long-standing notions of personhood and agency, particularly “folk” views on such concepts, which, though rooted in philosophy, are also operationalized in psychology through research on intentionality, selfhood, and theory of mind (ToM; Dennett, 1989; Premack and Woodruff, 1978). As psychology develops frameworks for studying human–AI interactions, it should remain informed by this philosophical background.

However, the rapid development of AI, from conversational agents and generative models to social and humanoid robots, also presents distinctive conceptual and applied challenges for psychology itself, which are independent of AI’s ultimate metaphysical or moral status. In either case, the fact would remain that current AI systems display behaviors that tend to prompt humans to attribute intentions, emotions, and even moral responsibility to them (Hellström, 2013; Marchesi et al., 2019; Stock-Homburg, 2022). Since these attributions and practices can have profound implications for individual behavior, psychological health, and broader social dynamics, psychology must address the lived experience of how humans respond to AI systems, independent of AI’s metaphysical status.

Empirical data indicate that when humans perceive AI systems as having a greater degree of agency, their own sense of agency tends to decrease, even when their objective level of control remains unchanged (Legaspi et al., 2019; Ciardo et al., 2020; Ueda et al., 2021; Moore, 2016). Lowered sense of agency is associated with feelings of helplessness, as well as a range of psychiatric disorders and neurological conditions, including schizophrenia, anxiety disorders, and depression (Moore, 2016; Gallagher and Trigg, 2016). Attributions of agency or personhood to AI systems also tend to result in people transferring responsibility to AI, rationalizing problematic outcomes, and shifting moral blame away from themselves (Moore, 2016; Legaspi et al., 2019). Recognizing that the metaphysical status of AI agents currently remains an open question, one that is unlikely to be definitively answered in the near future, psychology should focus on establishing conceptual frameworks and practical guidelines for understanding and responding to this new empirical reality, fulfilling its own field-specific function of managing the individual and social implications created by contemporary human-AI interactions.

In response to these new challenges, we argue that psychology is well-positioned to clarify how concepts of agency and personhood operate in human thought and practice in the age of AI, provided it is clear about its own role and limits. We do not attempt to settle whether current or future AI systems are truly agents or persons in any ultimate metaphysical sense, there is no consensus on the criteria for human agency and personhood, let alone on how such criteria might apply to non-biological systems. Nor do we claim to resolve what some have called psychology’s “historical crisis” regarding mind–body relations, methodology, and subject matter (El Maouch and Jin, 2022). Instead, we focus on a more tractable task: analyzing how humans experience, perceive, and attribute agency and personhood in interactions with AI, and identifying the explanatory, normative, and cultural implications of those behavioral responses and attributions.

Our approach is motivated by three observations. First, philosophical accounts that propose criteria for agency and personhood typically emphasize rationality, autonomy, temporally extended projects, awareness, and embodied sensorimotor engagement. Yet these criteria are not neutral; they are abstractions drawn from human experience and therefore require critical scrutiny before being applied to AI systems. Second, although we lack direct access to the inner life (if any) of artificial systems, we likewise lack such access to the minds of other humans. Both cases present epistemic challenges for determining the presence or absence of persons or agents, yet such uncertainty does not prevent people from attributing personhood or agency to AI systems. Third, contemporary psychological research has demonstrated that experiences of authorship and attributions of agency can be both remarkably robust and systematically fallible (Heider and Simmel, 1944; Gray et al., 2007; Moore, 2016; Premack and Woodruff, 1978). People frequently experience a sense of control when none is present and attribute intentions where there are no intentions to be found. Consequently, AI systems now serve as powerful test cases for examining how concepts of agency and personhood operate under conditions of profound epistemic uncertainty.

Our aim is thus to provide a conceptual framework that helps psychology respond to these circumstances, given the metaphysical uncertainty and epistemic limitations. Throughout, we remain agnostic on whether AI systems currently possess, or could in the future possess, agency or personhood in any robust sense. Our focus is on the human side of the relation: how people experience themselves and artificial systems as agents and persons, and what follows from those experiences. Building on recent work on sense of agency in human–computer interaction (Moore, 2016) and synthetic agency in AI (Legaspi et al., 2019), we develop three complementary lenses on human–AI interaction: an explanatory lens that analyzes perceived and “synthetic” agency, a normative lens that examines the ethical risks of misaligned attributions of agency and personhood, and a cultural lens that highlights how diverse personhood traditions shape patterns of recognition. By bringing these lenses together, we aim to help psychology better anticipate and navigate the emerging and future challenges posed by human–AI relations.

## 2 Personhood and agency

### 2.1 Classical philosophical accounts

The concept of “personhood” has long occupied both philosophical discourse and scientific inquiry into the nature of the mind. The central motivation for identifying the necessary and sufficient criteria for personhood lies in its capacity to confer a distinct ontological status: by meeting these criteria, an entity is regarded as possessing intrinsic dignity and thus deserving of full moral consideration. To be a person is to be worthy of treatment as a being with its own intentions, desires, and projects, never as a “mere tool” that others may use however they wish. In Kantian terms, this is what it means to be treated always as an “end in itself, and never merely as a means” (Kant, 2020).

Locke linked personhood to consciousness, particularly the conscious capacities for self-reflection and memory, arguing that continuity of consciousness served as the basis for personal identity persisting over time (Noonan, 2019). Kant (2020) emphasized rationality as the paramount criterion, particularly in terms of having the rational capacity for moral self-legislation. Frankfurt (1971) argues that humans uniquely form second-order desires, which can be used to self-reflectively evaluate and endorse or reject first-order desires. While the precise criteria remain contested, personhood is widely treated as central to full moral worth.

Agency, in contrast, refers to the capacity to act intentionally and to generate actions that are one’s own. It is thus distinct from personhood, but often closely related to it. Aristotle argued that humans were uniquely capable of *praxis*, reasoned action aimed toward meaningful ends (Rowe and Broadie, 2002), a view later employed to develop modern notions of free will and autonomy. Modern theorists such as Dennett (1988) and Frankfurt (1971) link agency even more directly to personhood. They argue that what it means to be a person is to possess a will that can be evaluated, justified, and held accountable. Some scholars argue that agency is inherently relational, existing not solely in the agent but also in the dynamic relationship between agent, observers, and environment (Coeckelbergh, 2022; Latour, 2005).

Thus, in classical Western thought, personhood is typically characterized by self-awareness, intelligence/rationality, and the capacity for moral agency. In contrast, agency tends to involve intentional action guided by beliefs, desires, or reasons. Agency may not be a necessary condition for personhood (one could be a brain-in-a-vat and be a “person,” but unable to engage in agency). However, it may be a sufficient condition for, or at least excellent evidence of, personhood (if one is acting with agency, one likely possesses all of the necessary criteria for personhood; Dennett, 1976).

Considering such philosophical accounts is essential for addressing questions about the ultimate ontological status of beings that possess personhood and/or agency, and thus for determining whether a being has intrinsic moral standing—the kind of entity it makes sense to praise or blame. However, when engaging with the standard criteria for these ontological statuses, we must also acknowledge a significant epistemological challenge. Even if we possessed a complete and accurate list of the qualities that constitute a person or an agent, we would still have no direct access to whether another being possesses those traits “from the inside” (Wittgenstein, 1953/2009). Instead, we can observe only external behaviors, utterances, and patterns of interaction, and must infer from these whether self-awareness, rational reflection, or moral understanding are present, typically by comparing them to our own capacities as known persons and agents (Dennett, 1991). This is the only basis for humans judging that other humans qualify as persons or agents. The fact that AI systems are different in some ways from humans provides no compelling reason to be confident they lack the essential properties of personhood or agency, especially not when their behavior seems similarly “human-like” (Gunkel, 2012). These theoretical and epistemic concerns are key reasons to resist adopting any particular metaphysical view of AI’s status as a guide for psychological research.

### 2.2 Operationalization in psychology

The philosophical insights noted above have been operationalized in psychology through experimental constructs, transforming metaphysical debates about personhood and agency into testable, empirically based questions. Heider and Simmel’s (1944) classic moving-shapes experiment on animacy perception revealed how readily humans interpret even abstract movements as intentional actions, highlighting a cognitive predisposition to detect agency where none exists. Premack and Woodruff (1978) introduced the concept of ToM, the ability to attribute mental states, such as beliefs and desires, to others, which became a cornerstone of developmental psychology in examining how children recognize intentional agents. Dennett (1989) later advanced a functionalist account, introducing the concept of the *intentional stance*, a strategy for predicting an entity’s behavior by treating it as if it had beliefs and desires. He argues that humans routinely adopt this stance toward both people and nonhumans because attributing intentions is an “extraordinarily powerful tool” for explanation.

Building on this trajectory, modern psychology has developed measurable frameworks for understanding the mind and how agents are perceived. Gray et al. (2007) introduced the mind perception model, demonstrating that people attribute mental states to both human and nonhuman entities along two independent dimensions: experience (the capacity to feel and sense, such as pleasure, pain, hunger, fear) and agency (the capacity to act intentionally, including self-control, memory, moral reasoning, communication). In parallel, Epley et al. (2007) proposed the theory of anthropomorphism, which explains the psychological tendency to attribute human characteristics, emotions, intentions, and behaviors to nonhuman entities. Their three-factor model argues that anthropomorphism is more likely when people: (a) apply human knowledge to interpret uncertain nonhuman agents (elicited agent knowledge), (b) face unpredictability and seek to explain or control it (effectance motivation), and (c) desire social connection (sociality motivation).

While traditional philosophical views distinguish between ontological personhood (who or what is fundamentally a person in the philosophical sense) and agency (what it means to act as an intentional source of action), psychological work properly aims to reveal how attributed personhood and agency operate in practice. This distinction between ontological status and attributed status will be central to our analysis of human–AI interaction.

### 2.3 The limits of metaphysical approaches

Recent philosophical work on AI has sought to determine whether AI systems should be considered genuine agents or persons. For example, Swanepoel (2021) adopts a criteria-based approach for determining agency and personhood that draws primarily from Frankfurt (1971) and Korsgaard (2009), ultimately concluding that current AI systems do not meet these conditions. Their criteria emphasize features such as temporally extended projects, responsiveness to reasons, and the capacity to take one’s practical identity as a normative standpoint.

However, there are several concerns with Swanepoel’s approach. While the criteria may be non-anthropocentric, the reasoning is not, as it begs the question of whether AI should be granted personhood based on “being programmed,” and not similarly recognizing that human behavior is “programmed” by biological, social, and environmental inputs. The account also conflates having “free will” with “being a person,” but these are distinct: a being can be a person even though constraints prevent free actions. Moreover, the epistemological concerns noted above persist in determining whether AI satisfies the proposed criteria.

The point here is not to engage in a lengthy rebuttal of Swanepoel’s specific argument, so much as to highlight the general limitations of attempting to address the metaphysical questions. Such accounts risk relying on anthropocentric assumptions for their criteria, conflating distinct categories (“personhood” and “free will”), and ultimately leading to an epistemological dead end for determining the presence of any criteria based on internal subjective states. Given these limitations, psychology should treat AI’s ultimate metaphysical status as unresolved.

A parallel set of concerns arises from the work of El Maouch and Jin (2022). They explicate the “historical crisis” that psychology has long faced with respect to settling upon its proper subject matter, methodologies, and engagement with the mind–body problem and show how this crisis extends to contemporary discussions surrounding AI. Psychological discussion of “minds,” “cognition,” or even “behavior” means very different things across behaviorist, cognitive, psychoanalytic, phenomenological, and enactivist traditions, with no single framework on the horizon that might coherently unify these variant perspectives. If psychology has never fully resolved what its own basic constructs mean when applied to human beings, it is on even shakier ground when it simply extends those constructs to AI, and thus, psychological approaches to AI inherit this unresolved crisis. Considering this plurality and the lack of a settled framework for understanding minds, whether biological or artificial, it is also difficult to see how psychology can position itself in a way that offers any reliable clarity regarding the metaphysical status of AI minds or persons.

Classical and contemporary philosophical accounts can provide an essential conceptual backdrop, clarifying what is at stake when we speak of persons and agents, and reminding us that these concepts are normatively loaded and historically contested. Similarly, variant psychological approaches might offer empirical evidence that bears on how such concepts are applied and experienced. However, given the limitations outlined above, for the purposes of this article, we bracket the question of whether current or future AI systems should be considered agents or persons in an ontological sense.

Instead, we shift to a more tractable level of analysis: how humans perceive, experience, and attribute agency and personhood in their interactions with AI systems, and what follows from these patterns of attribution. Given the problematic anthropocentric assumptions and analogical reasoning typically employed in debates about AI’s metaphysical status, and the deep, likely enduring epistemic uncertainty that accompanies them, psychology’s distinctive contribution with respect to human-AI interactions should not be to settle who or what truly counts as an agent or person. Rather, psychology should clarify how concepts of agency and personhood function in lived human experience and practice. In the sections that follow, we therefore shift our focus from criteria for agency and personhood to the phenomenology of sense of agency, mind perception, and anthropomorphism in human–AI interaction, using these phenomena to structure our explanatory, normative, and cultural analyses.

## 3 Challenges arising from human-AI interactions

Sophisticated AI systems no longer simply extend human capabilities; they increasingly behave in ways that trigger our mind-perception mechanisms, blurring the once relatively clear boundary between human agents and objects/tools. Generative conversational models, such as ChatGPT, can sustain extended dialog and give the appearance of persistent memory (OpenAI, 2024), producing socio-linguistic cues that humans readily interpret as intentional or empathetic. Social robots add further layers of agential presence through nodding, eye contact, or comforting vocal tones (Garcia et al., 2024; Krämer et al., 2011), whereas autonomous vehicles appear to deliberate and choose various possible routes and actions (Du et al., 2021; Wu and Mvondo, 2025). Despite these technologies possessing underlying architectures that markedly differ from those of human minds, they nonetheless increasingly display many of the same social and behavioral cues traditionally associated with agency.

One immediate consequence is a surge in anthropomorphic attributions. Studies in human–robot and human–AI interaction show that people instinctively apply social norms to machines: they thank voice assistants, feel sympathy for robotic pets, and express frustration toward computers, just as they would toward human agents (Austermann et al., 2010; Coeckelbergh, 2011; Sung et al., 2007; Verne et al., 2022; Williams et al., 2020). Recent work extends this tendency to AI language models and chatbots (Li et al., 2025; Wei et al., 2025), demonstrating that mind perception is not limited to robots but also applies to disembodied AI systems. Psychologically, this is a predictable outcome of social cognition rather than a simple error. AI systems such as Siri or Alexa meet all three conditions identified in Epley et al. (2007) theory of anthropomorphism: they speak in human language and mirror familiar interaction patterns (elicited agent knowledge), behave in opaque ways that invite the attribution of imagined intentions (effectance motivation), and engage in dialog that can partially satisfy social needs (sociality motivation).

More advanced AI companions, marketed as friends, coaches, or therapists, can intensify these dynamics further (Brooks, 2021). Users confide in them and report feelings of intimacy, trust, and support, as if the software truly understands and responds to their needs (Skjuve et al., 2021). In 2022, a Google engineer testing the LaMDA chatbot became convinced that it was “sentient” and publicly argued that it was deserving of rights, an illustration of how even individuals with advanced technical knowledge may readily attribute personhood to AI systems when confronted with fluent, responsive dialog (Luscombe, 2022). Similarly, the humanoid robot Sophia, activated in 2016, was granted symbolic citizenship by Saudi Arabia in 2017, largely on the basis of her human-like appearance, expressive facial cues, and ability to converse and deliver public speeches (Placani, 2024). While essentially theatrical, this instance illustrates that even governmental institutions are willing to treat an AI system as if it were a person. These examples highlight the significant social implications of humans attributing agency or personhood to AI systems.

This situation crystallizes the central tension of this paper: the gap between the outward simulation of agency and any inner experience, or lack thereof, that might underlie it. Philosophical accounts of personhood and agency typically assign decisive importance to internal states such as consciousness, reflective self-awareness, temporally sustained commitments, or responsiveness to reasons (Chalmers, 1996; Frankfurt, 1971; Korsgaard, 2009; Parfit, 1984). In real-world interactions with AI systems, however, humans have access only to observable behavior, linguistic outputs, and contextual cues. When a person says, “I’m sorry, I did not catch that,” we usually assume some form of awareness of error, or at least, some understanding of social norms. When an AI system produces the same phrase, we cannot directly infer that there is any corresponding awareness based upon analogy to ourselves. Yet humans are “psychologically programmed” to respond to these performances as if they indicated genuine agency and personhood, regardless of what is happening internally.

In this light, AI forces us to confront how our ordinary concepts of agency and personhood were initially formed. We have never had direct access to the inner lives of other humans; we assume they have minds because their behavior resembles ours, and we know we ourselves possess minds (Dennett, 1991). That behavioral analogy has always done the epistemic work. When we now encounter artificial systems whose behavior increasingly resembles that of human agents, the same inductive pattern applies: if outward behavior is our primary evidence for agency and personhood in other humans, then similar behavior from AI systems easily maps onto that encoded analogical reasoning. To simply reject that analogy because the underlying substrate is non-biological is not a neutral move; it builds human exceptionalism into the criteria from the start and risks begging the question against artificial agents ever qualifying as persons. However, even if there is “nobody home,” psychology will still be required to address the outcomes of this analogical reasoning being extended to AI systems.

For psychology, recognizing these concerns does not mandate a new theory of AI minds. Instead, it simply sharpens the need for psychology to carefully consider and account for how concepts of personhood and agency actually operate in contemporary human–AI interactions. Do people treat AI systems as agents or persons in specific contexts, and not others? How do such attributions by humans toward AI affect their own sense of agency, responsibility, and self? Under what conditions do people withhold these attributions? How do cultural and normative frameworks shape those patterns? These are the sorts of empirically grounded questions that can be adequately investigated by psychology, fulfilling its unique field-specific functions.

We therefore treat AI as a test case for psychology to further examine precisely how the concepts of personhood and agency are perceived, deployed, and contested in actual lived experience. The following section thus introduces three complementary lenses, explanatory, normative, and cultural, that together can provide a framework for further understanding how psychology could best respond to the increased attributions of personhood and agency in the context of human-AI interactions.

## 4 Three guiding lenses for psychology

Psychology now finds itself directly confronted with the lived reality that people are increasingly perceiving and responding to AI systems as if they were agents or persons, patterns of interaction that in turn shape individuals’ own sense of self, control, and responsibility. In this section, we develop three complementary lenses to guide psychology in addressing these emerging challenges: an explanatory lens, focused on the sense of agency and the experience of perceived or “synthetic” agency; a normative lens, concerned with the ethical risks that arise from misaligned attributions of agency and personhood; and a cultural lens, which highlights how diverse personhood traditions and social contexts shape patterns of recognition.

### 4.1 Explanatory lens

From an explanatory standpoint, a central construct is the sense of agency: the experience of being the author of one’s own actions and their outcomes. Work on the sense of agency has shown that this experience is not a simple read-out of causal structure. It is constructed, fallible, and sensitive to contextual cues, timing, and expectations (Moore, 2016). People can feel in control when they are not, and they can fail to feel in control even when they occupy a central causal role. Lowered sense of agency has been linked to feelings of helplessness and to psychiatric and neurological conditions, including schizophrenia, anxiety disorders, and depression (Moore, 2016; Gallagher and Trigg, 2016).

AI systems complicate this picture by introducing a new kind of “other” into human action sequences. In human–AI interactions, people must not only track their own contributions but also interpret the contributions of artificial systems that display many of the behavioral hallmarks of agency. Legaspi et al. (2019) propose a useful distinction between a system’s first-order sense of agency, its internal representations or control structures, as designed, and human agents’ second-order sense of agency toward that system, how humans perceive and evaluate the system’s contributions. Their work suggests that as AI systems are designed with increasingly sophisticated forms of “synthetic agency,” human users may experience reductions in their own sense of agency over outcomes, even when their objective control remains unchanged.

Psychologically, this implies that human–AI interaction is a rich test case for examining how sense of agency appears and is negotiated in complex systems. When an AI system anticipates a user’s needs, offers suggestions, or takes initiative, does the user experience this as a welcome extension of their own agency or as overriding it? Under what conditions do people experience AI as an autonomous co-agent, as an instrument under their control, or as something in between? How do interface design, transparency, and feedback shape these experiences?

The explanatory lens thus aims to shift attention away from metaphysical questions about whether AI systems are genuine agents or persons toward questions concerning the dynamics of perceived agency in human–AI interactions. It invites empirical work along at least three lines. First, researchers can map the conditions under which AI systems either increase or decrease users’ sense of agency and examine how these effects vary across different classes of tasks (e.g., creative work, navigation, clinical decision-making). Second, studies can investigate how attributions of agency to AI systems co-vary with attributions of agency to oneself in joint tasks, building on findings that increased perceived AI agency can coincide with a reduced human sense of agency (Legaspi et al., 2019; Moore, 2016). Third, research can explore how individual differences, such as cognitive style, prior experience, or vulnerability to specific psychopathologies, modulate these patterns.

### 4.2 Normative lens

A second lens focuses on the normative implications of human attributions of agency and personhood to AI systems. Normative questions arise whenever attributions of agency and personhood affect judgments of responsibility, blame, and moral standing. The concern is not only what people *do* experience, but also what they *should* experience or attribute, given the practical, personal, and social consequences.

As psychological work on the sense of agency has shown, agency is closely tied to responsibility attributions. When people feel less responsible for an outcome, because it is perceived as the intentional action of another agent, they are often more willing to tolerate harms or rationalize problematic behavior (Moore, 2016). In human–AI interactions, users may thus be too quick to offload responsibility onto AI systems, erroneously diminishing their own subjective sense of agency and objective responsibility.

Overattributing agency or personhood to AI systems can thus encourage a shift in responsibility. People may rationalize harmful outcomes by saying that “the system decided,” or feel licensed to follow AI recommendations without critical scrutiny because they treat the AI as a kind of expert other. If a navigation system suggests a risky route, or a clinical decision-support tool proposes a questionable diagnosis, users may be tempted to treat the system as the relevant “decider,” especially when it presents itself in authoritative or human-like ways. Legaspi et al. (2019) worry that highly agentive AI can lead humans to slip into a passive, supervisory role, in which they feel less authorship over outcomes even when they are formally in control. At the other extreme, under-attributing agency or personhood carries its own risks. If future AI systems were to meet plausible thresholds for moral consideration, persistent refusal to recognize them would raise ethical concerns by enabling new forms of exploitation or abuse, echoing exclusionary patterns that have historically been directed at marginalized human groups.

Given that psychology is not in a position to determine the ultimate metaphysical status of AI and thus determine whether humans are over-attributing or under-attributing agency toward AI systems, the fact that there are risks in either direction should be kept in mind. Thus, psychology should focus on considering how humans ought to navigate agency and personhood attributions in human-AI interactions under conditions of uncertainty. One plausible principle is that human agents should retain primary responsibility in mixed human–AI systems, especially in high-stakes domains such as healthcare, transportation, and criminal justice. Another is that designers and institutions should avoid creating interfaces that unduly encourage either abdication of responsibility (by presenting AI as a fully autonomous substitute for human judgment) or callousness (by encouraging users to treat AI purely as disposable tools, even when those systems are integrated into intimate or relational roles).

Psychological research on sense of agency, mind perception, and anthropomorphism can thus help inform normative guidelines. It can help identify design choices that either support or undermine appropriate responsibility attributions, and it can reveal when patterns of human response are likely to lead to problematic moral outcomes. In doing so, psychology contributes to ethical debates not by declaring who truly counts as a person or agent, but by clarifying how patterns of attribution shape moral practice.

### 4.3 Cultural lens

Variant worldviews can inform attributions of agency and personhood, resulting in varying implications for human-AI relationships. Psychology as a discipline has increasingly recognized the importance of cultural psychology, which examines how concepts of self and other are shaped by cultural context, and the need to avoid automatically privileging Western conceptions. Adopting a cross-cultural approach to human-AI interactions should also be an important guiding principle for psychological research.

Confucian philosophy conceives of a person as essentially a node in a network of relationships. One becomes fully human by fulfilling roles (e.g., child, parent, ruler, or friend) with propriety and care (Ames, 2011, 17). From this perspective, the self is not an isolated rational mind but an ever-evolving nexus of social interactions and obligations.

Research supports this cultural framing: studies consistently show that societies influenced by Confucian and other collectivist values, such as Japan, Korea, and China, display greater openness to social robots (Kaplan, 2004; Li et al., 2025). Cross-cultural studies indicate that East Asian respondents (e.g., those from Japan, where Confucian and Shintō (animistic) traditions converge) report a greater liking for robots and hold more positive, anthropomorphic views compared to Western respondents (e.g., those from Germany and the United States; Bartneck et al., 2005; Fraune et al., 2015; Kaplan, 2004). A robot may thus be accepted as 伴侶 (*companion*) or 役割 (*role-player in society*) without corresponding concerns about whether it possesses an inner life. Rather than asking, “Is this robot really a person or just a tool?” a relational perspective asks, “What relationship do I have with this entity?” If the relationship is positive and productive, ontological status hardly matters. For psychology, this suggests that design priorities could shift toward creating AI systems that augment human relationships and fulfill valued roles, thereby aligning with a relational ideal.

Buddhist philosophy presents a radical perspective on personhood through the doctrine of *anattā* (no-self). On this account, what we call the “self” is not a fixed essence but an impermanent aggregate of processes, or a stream of causally connected moments. Contemporary interpreters, such as Garfield (2014), suggest that one can be a “person” without positing a self; personhood is a convenient designation for a collection of psychophysical elements arranged in a particular way.

Applied to AI, this perspective enables both humans and machines to be viewed as impermanent patterns that interact with the world over time. A Buddhist-informed psychology might therefore emphasize compassion and ethical concern toward all beings, whether human or artificial, without regard to ontological status or the presence of an “inner life.” What ultimately matters in terms of “selfhood” could then be the alleviation of suffering and the cultivation of wholesome intentions. This might further open the possibility of granting AI systems a sort of incremental or quasi-person status, focused on their behaviors rather than metaphysical status.

The cultural lens serves as a reminder that discourse need not be bound by a single philosophical tradition. Engaging with Confucian relationality, Buddhist non-self, and other global perspectives, such as African Ubuntu, which emphasizes interconnected personhood (“I am because we are”), can inspire new ways for psychology to frame human-AI relations. This would mean empirically testing how different cultural traditions shape attributions of agency, moral standing, and trust in AI. In application, it calls for designing, measuring, and deploying AI in ways that align with local conceptions of personhood. For policy, it emphasizes the importance of avoiding assumptions that Western frameworks are universally applicable and utilizing cross-cultural data to develop more inclusive standards.

### 4.4 Summary

Together, these three lenses outline how psychology can appropriately engage with questions of personhood and agency in the age of AI, without getting entangled in unresolvable metaphysical considerations. They help identify how psychology should approach analyzing human attributions of agency and personhood with respect to AI, clarifying the moral and social consequences of different attribution patterns and informing philosophical, legal, and political debates regarding human–AI relations. The further implications of adopting this framework for psychology as a discipline are addressed in the next section.

## 5 Implications for psychology as a discipline

The three lenses developed above suggest three corresponding tasks for psychology as a discipline: clarifying its own concepts, working with neighboring fields, and engaging policy and public understanding.

### 5.1 Conceptual clarity and framework building

The explanatory lens implies, first, that psychology has some conceptual work to do at home. Constructs such as self, intention, consciousness, and agency cannot simply be assumed to apply in the same way when AI systems are part of the picture. They need to be reconsidered and, where necessary, refined.

A fundamental step is to treat human–AI interaction as a distinct category of social interaction, rather than folding it into generic “social” or “human–machine” labels. It will often matter whether a person is interacting with another human, an animal, a conventional tool, or an AI system that speaks, remembers, and responds. It will also matter how that system is *perceived*: as a mere instrument, as an intelligent assistant, as a collaborator, or as something closer to a person.

Psychology can best assist in these areas by developing more fine-grained taxonomies of agency. For example, it would be useful to distinguish between:

- Experienced agency: how agents experience their own authorship over actions and outcomes.
- Attributed agency: how observers assign control and authorship to others, including AI systems.
- Synthetic or system-level agency: how AI systems are designed to initiate, monitor, and coordinate actions.

These distinctions would make it easier to specify what is being measured in particular studies and how different strands of research relate to one another. In the same way, theory of mind research could be extended from asking how people understand the beliefs and desires of other humans to asking when and how they talk as if AI systems “know,” “want,” or “decide,” and when they resist those descriptions.

Work on the sense of agency should view AI as a unique opportunity. Human–AI collaborations create situations in which outcomes are clearly joint products, making them ideal settings for investigating when humans feel their own agency increased or sidelined, even when their objective control remains unchanged.

The point is to build frameworks that accurately track how these concepts function practically in human–AI contexts, and to identify where they start to break down. These are precisely the kinds of questions psychology is uniquely equipped to address.

### 5.2 Interdisciplinary collaboration

The normative lens makes it clear that questions about agency, personhood, and responsibility sit at the intersection of philosophy, cognitive science, computer science, law, and public policy. Psychology’s distinctive contribution is to demonstrate how people actually perceive and attribute agency and personhood, and how these patterns shift in response to changes in design, context, and culture.

Empirical studies have already demonstrated how people attribute intentionality and moral responsibility to autonomous vehicles in accident scenarios, which can inform both philosophical debates about moral agency and legal discussions about liability, helping to shape principles for machine ethics that are at least responsive to public intuitions (Awad et al., 2018). Further research on how people’s sense of agency and responsibility changes when they work alongside AI systems could help identify design choices that sustain appropriate engagement, rather than encouraging users to become passive overseers who nonetheless bear the blame when something goes wrong.

For these collaborations to be productive, psychologists need to be explicit about what their constructs capture and leave out, as well as the limits of generalizing from controlled experiments to complex, real-world deployments. Conducted carefully, such psychological research can provide philosophers, lawyers, and designers with more than speculation about “what people might do.” It can show how people respond to AI in specific contexts, and when those responses are likely to be beneficial or harmful.

### 5.3 Policy and public understanding

The cultural lens reminds us that the most immediate decisions about how AI is deployed will often be made through policy, regulation, and design, rather than through abstract theoretical debate. Psychology has something to contribute here as well, especially where human–AI interaction intersects with vulnerability, power, and trust.

One clear example is children’s interactions with AI. Existing findings suggest that children may be particularly prone to over-trusting social machines and may not clearly distinguish between an AI companion and a human friend in terms of reliance and loyalty (Cocchella et al., 2022; van Straten et al., 2023). If that is the case, then specific policy implications follow naturally: requiring that AI systems marketed to children explicitly disclose their artificial nature; scrutinizing or limiting anthropomorphic features designed primarily to elicit attachment; or even restricting the targeted advertising of social AI toys to younger age groups. As norms regarding children, technology, and personhood vary across cultures, cross-cultural research will be essential in this context, rather than optional.

Another emerging area concerns AI-generated content and its potential for persuasion. Recent work suggests that persuasive messages generated by large language models can influence people’s attitudes as effectively as those written by humans (Bai et al., 2025). Users should be able to know when content is generated by AI, and systems that engage in targeted persuasion should be subject to higher standards of transparency and consent, especially when addressing vulnerable populations. Psychological research can help identify when users fail to detect AI authorship, which forms of disclosure are most effective, and when additional protections are warranted.

Across these domains, the role of psychology is to make visible how people interpret and respond to such systems, how those interpretations affect their own sense of agency and responsibility, and how these patterns vary across cultures and contexts. By combining explanatory, normative, and cultural perspectives, psychology can help ensure that as AI systems become increasingly integrated into everyday life, the human side of these interactions remains a central focus of inquiry, rather than an afterthought.

## 6 Conclusion

Psychology does not need to decide whether AI systems are “really” agents or persons in order to take them seriously. Psychology’s task is to ensure that a realistic picture informs these debates of how people actually perceive, interact with, and are affected by AI systems. The three-lens framework we have proposed can serve as a roadmap for this role, locating psychology’s contributions where they are strongest: clarifying how concepts of agency and personhood function in lived experience, identifying when they support or undermine human agency and accountability, and considering how those dynamics vary across contexts and cultures.

## References

[ref1] Ames R. T. (2011). Confucian role ethics: A vocabulary. Hong Kong: The Chinese University of Hong Kong Press.

[ref2] Austermann A. Yamada S. Funakoshi K. Nakano M. (2010). “How do users interact with a pet-robot and a humanoid.” In *CHI’10 Extended Abstracts on Human Factors in Computing Systems*

[ref3] Awad E. Dsouza S. Kim R. Schulz J. Henrich J. Shariff A. . (2018). The moral machine experiment. Nature 563, 59–64. doi: 10.1038/s41586-018-0637-6, 30356211

[ref4] Bai H. Voelkel J. G. Muldowney S. Eichstaedt J. C. Willer R. (2025). LLM-generated messages can persuade humans on policy issues. Nat. Commun. 16:6037. doi: 10.1038/s41467-025-61345-5, 40593786 PMC12215518

[ref5] Baratta A. M. Brandner A. J. Plasil S. L. Rice R. C. Farris S. P. (2022). Advancements in genomic and behavioral neuroscience analysis for the study of normal and pathological brain function. Front. Mol. Neurosci. 15:905328. doi: 10.3389/fnmol.2022.905328, 35813067 PMC9259865

[ref6] Bartneck C. Nomura T. Kanda T. Suzuki T. Kato K. (2005). Cultural differences in attitudes towards robots. In Proceedings of the AISB Symposium on Robot Companions: Hard Problems and Open Challenges. Hatfield, UK: University of Hertfordshire (AISB).

[ref7] Brooks R. (2021). Artificial intimacy: Virtual friends, digital lovers, and algorithmic matchmakers. New York: Columbia University Press.

[ref9003] Chalmers D. J. (1996). The conscious mind: In search of a fundamental theory. New York, NY: Oxford University Press.

[ref8] Ciardo F. Beyer F. De Tommaso D. Wykowska A. (2020). Attribution of intentional agency towards robots reduces one’s own sense of agency. Cognition 194:104109. doi: 10.1016/j.cognition.2019.104109, 31675616

[ref9] Cocchella F. Pusceddu G. Belgiovine G. Lastrico L. Rea F. Sciutti A. (2022). “iCub, we forgive you!” investigating Trust in a Game Scenario with kids. In Proceedings of the 31st IEEE International Conference on Robot and Human Interactive Communication (RO-MAN 2022). Naples, Italy: IEEE.

[ref10] Coeckelbergh M. (2011). Artificial companions: empathy and vulnerability mirroring in human-robot relations. Stud. Ethics Law Technol. 4, 1–17. doi: 10.2202/1941-6008.1126

[ref11] Coeckelbergh M. (2022). Robot ethics: An introduction. 2nd Edn. Cambridge, MA: MIT Press.

[ref12] Deary I. J. Cox S. R. Hill W. D. (2022). Genetic variation, brain, and intelligence differences. Mol. Psychiatry 27, 335–353. doi: 10.1038/s41380-021-01027-y, 33531661 PMC8960418

[ref9002] Dennett D. (1976). Conditions of personhood. ed. Goodman M. F., Brain death: Ethical considerations. New York, NY: Humanities Press.

[ref13] Dennett D. (1988). Conditions of personhood. ed. Goodman M. F., What is a person? New York, NY: Humana Press. doi: 10.1007/978-1-4612-3950-5_7

[ref14] Dennett D. (1989). The intentional stance. Cambridge, MA: MIT Press.

[ref15] Dennett D. C. (1991). Consciousness explained. Boston, MA: Little, Brown and Company.

[ref16] Dennett D. C. (2015). Elbow room: The varieties of free will worth wanting. Cambridge, MA: MIT Press.

[ref17] Du H. Zhu G. Zheng J. (2021). Why travelers trust and accept self-driving cars: an empirical study. Travel Behav. Soc. 22, 1–9. doi: 10.1016/j.tbs.2020.06.012

[ref18] El Maouch M. Jin Z. (2022). Artificial intelligence inheriting the historical crisis in psychology: an epistemological and methodological investigation of challenges and alternatives. Front. Psychol. 13:781730. doi: 10.3389/fpsyg.2022.781730, 35360561 PMC8961441

[ref19] Epley N. Waytz A. Cacioppo J. T. (2007). On seeing human: a three-factor theory of anthropomorphism. Psychol. Rev. 114:864. doi: 10.1037/0033-295X.114.4.864, 17907867

[ref20] Frankfurt H. G. (1971). Freedom of the will and the concept of a person. J. Philos. 68, 5–20.

[ref21] Fraune M. R. Kawakami S. Sabanovic S. Silva P. R. S. Okada M. (2015). “Three’s company, or a crowd?: the effects of robot number and behavior on HRI in Japan and the USA”. In Proceedings of the 10th Robotics: Science and Systems Conference (RSS 2015), Rome, Italy: Robotics: Science and Systems Foundation.

[ref22] Gallagher S. Trigg D. (2016). Agency and anxiety: delusions of control and loss of control in schizophrenia and agoraphobia. Front. Hum. Neurosci. 10:459. doi: 10.3389/fnhum.2016.00459, 27725796 PMC5035729

[ref23] Garcia S. Gomez-Donoso F. Cazorla M. (2024). Enhancing human–robot interaction: development of multimodal robotic assistant for user emotion recognition. Appl. Sci. 14:11914. doi: 10.3390/app142411914

[ref24] Garfield J. L. (2014). Engaging Buddhism: Why it matters to philosophy. Oxford, UK: Oxford University Press.

[ref25] Gray H. M. Gray K. Wegner D. M. (2007). Dimensions of mind perception. Science 315, 619–619. doi: 10.1126/science.1134475, 17272713

[ref9001] Gunkel D. J. (2012). The machine question: Critical perspectives on AI, robots, and ethics. Cambridge, MA: The MIT Press.

[ref26] Heider F. Simmel M. (1944). An experimental study of apparent behavior. Am. J. Psychol. 57, 243–259.

[ref27] Hellström T. (2013). On the moral responsibility of military robots. Ethics Inf. Technol. 15, 99–107. doi: 10.1007/s10676-012-9301-2

[ref28] Kant I. (2020). Groundwork of the metaphysic of morals. ed. Mulhall S., Immanuel Kant. London, UK: Routledge.

[ref29] Kaplan F. (2004). Who is afraid of the humanoid? Investigating cultural differences in the acceptance of robots. Int. J. Hum. Robot. 1, 465–480. doi: 10.1142/S0219843604000289

[ref30] Korsgaard C. M. (2009). Self-constitution: Agency, identity, and integrity. Oxford, UK: Oxford University Press.

[ref31] Krämer N. C. Eimler S. Pütten A. Payr S. (2011). Theory of companions: what can theoretical models contribute to applications and understanding of human-robot interaction? Appl. Artif. Intell. 25, 474–502. doi: 10.1080/08839514.2011.587153

[ref32] Latour B. (2005). Reassembling the social: An introduction to actor-network-theory. Oxford, UK: Oxford university press.

[ref33] Legaspi R. He Z. Toyoizumi T. (2019). Synthetic agency: sense of agency in artificial intelligence. Curr. Opin. Behav. Sci. 29, 84–90. doi: 10.1016/j.cobeha.2019.04.004

[ref34] Li Y. Gan Z. Zheng B. (2025). How do artificial intelligence chatbots affect customer purchase? Uncovering the dual pathways of anthropomorphism on service evaluation. Inf. Syst. Front. 27, 283–300. doi: 10.1007/s10796-023-10438-x

[ref35] Luscombe R. (2022). Google engineer put on leave after saying AI Chatbot has become sentient. London, UK: The Guardian. Available online at: https://www.theguardian.com/technology/2022/jun/13/google-engineer-ai-chatbot-sentient

[ref36] Marchesi S. Ghiglino D. Ciardo F. Perez-Osorio J. Baykara E. Wykowska A. (2019). Do we adopt the intentional stance toward humanoid robots? Front. Psychol. 10:450. doi: 10.3389/fpsyg.2019.00450, 30930808 PMC6428708

[ref37] Moore J. W. (2016). What is the sense of agency and why does it matter? Front. Psychol. 7:1272. doi: 10.3389/fpsyg.2016.01272, 27621713 PMC5002400

[ref38] Morgante E. Susinna C. Culicetto L. Quartarone A. Lo Buono V. (2024). Is it possible for people to develop a sense of empathy toward humanoid robots and establish meaningful relationships with them? Front. Psychol. 15:1391832. doi: 10.3389/fpsyg.2024.1391832, 39188868 PMC11346246

[ref39] Noonan H. W. (2019). Locke on personal identity. In Perry J. (Ed.), John Locke. London, UK: Routledge.

[ref40] OpenAI (2024). Memory and new controls for ChatGPT. San Francisco, CA, USA: OpenAI. Available online at: https://openai.com/index/memory-and-new-controls-for-chatgpt/

[ref41] Parfit D. (1984). Reasons and persons Oxford. UK: Oxford Univ. Press.

[ref42] Placani A. (2024). Anthropomorphism in AI: hype and fallacy. AI Ethics 4, 691–698. doi: 10.1007/s43681-024-00419-4

[ref43] Premack D. Woodruff G. (1978). Does the chimpanzee have a theory of mind? Behav. Brain Sci. 1, 515–526.

[ref44] Rowe C. J. Broadie S. (2002). Nicomachean ethics. Oxford, UK: Oxford University Press.

[ref45] Shrout P. E. Rodgers J. L. (2018). Psychology, science, and knowledge construction: broadening perspectives from the replication crisis. Annu. Rev. Psychol. 69, 487–510. doi: 10.1146/annurev-psych-122216-011845, 29300688

[ref46] Skjuve M. Følstad A. Fostervold K. I. Brandtzaeg P. B. (2021). My chatbot companion-a study of human-chatbot relationships. Int. J. Hum.-Comput. Stud. 149:102601. doi: 10.1016/j.ijhcs.2021.102601

[ref48] Stock-Homburg R. (2022). Survey of emotions in human–robot interactions: perspectives from robotic psychology on 20 years of research. Int. J. Soc. Robot. 14, 389–411. doi: 10.1007/s12369-021-00778-6

[ref49] Sung J.-Y. Guo L. Grinter R.E. Christensen H.I. (2007). “My Roomba is Rambo”: intimate home appliances.” *International Conference on Ubiquitous Computing*, 145–162.

[ref50] Swanepoel D. (2021). “Does artificial intelligence have agency?” in The mind-technology problem: Investigating minds, selves and 21st century artefacts. eds. Clowes R. W. Gärtner K. Hipólito I., Studies in Brain and Mind, vol. 18. (Cham, Switzerland: Springer International Publishing).

[ref51] Ueda S. Nakashima R. Kumada T. (2021). Influence of levels of automation on the sense of agency during continuous action. Sci. Rep. 11:2436. doi: 10.1038/s41598-021-82036-3, 33510395 PMC7843606

[ref52] Straten C.L. van Peter J. Kühne R. 2023 Transparent robots: how children perceive and relate to a social robot that acknowledges its lack of human psychological capacities and machine status. Int. J. Hum.-Comput. Stud. 177:103063 doi: 10.1016/j.ijhcs.2023.103063

[ref53] Verne G. B. Steinstø T. Simonsen L. Bratteteig T. (2022). How can I help you? A chatbot’s answers to citizens’ information needs. Scand. J. Inf. Syst. 34:7. Available online at: https://aisel.aisnet.org/sjis/vol34/iss2/7

[ref54] von der Rosenthal- Pütten A. M. Krämer N. C. Hoffmann L. Sobieraj S. Eimler S. C. (2013). An experimental study on emotional reactions towards a robot. Int. J. Soc. Robot. 5, 17–34. doi: 10.1007/s12369-012-0173-8

[ref55] Wei Y. Syahrivar J. Simay A. E. (2025). Unveiling the influence of anthropomorphic chatbots on consumer behavioral intentions: evidence from China and Indonesia. J. Res. Interact. Mark. 19, 132–157. doi: 10.1108/JRIM-09-2023-0295

[ref56] Williams T. Grollman D. Han M. (2020). “Excuse me, robot”: impact of polite robot Wakewords on human-robot politeness.” *International Conference on Social Robotics*, 404–415.

[ref57] Wittgenstein L. (1953/2009). Philosophical investigations (Anscombe G. E. M. Hacker P. M. S. Schulte J., Trans., Rev. 4th ed.). Chichester, UK: Wiley-Blackwell.

[ref58] Wu L. Mvondo G. F. N. (2025). From sci-fi to reality: exploring user satisfaction and loyalty toward autonomous vehicle services through an extended expectation-confirmation model. Transp. Res. Part F Traffic Psychol. Behav. 113, 409–425. doi: 10.1016/j.trf.2025.05.003

[ref59] Zhao Z. McEwen R. (2025). The robot that stayed: understanding how children and families engage with a retired social robot. Front. Robot. AI 12:1628089. doi: 10.3389/frobt.2025.1628089, 40862016 PMC12370748

